# Physical activity reverses the aging induced decline in angiogenic potential in the fast locomotory muscles of mice

**DOI:** 10.1038/s41598-025-93176-1

**Published:** 2025-03-14

**Authors:** Magdalena Zmudzka, Joanna Szramel, Janusz Karasinski, Zenon Nieckarz, Jerzy A. Zoladz, Joanna Majerczak

**Affiliations:** 1 Chair of Exercise Physiology and Muscle Bioenergetics, Faculty of Health Sciences, Jagiellonian University Medical College, Skawinska 8 Street, Krakow, 31-066 Poland; 2 Department of Cell Biology and Imaging, Institute of Zoology and Biomedical Research, Faculty of Biology, Jagiellonian University, Krakow, Poland; 3 Department of Experimental Computer Physics, Marian Smoluchowski Institute of Physics, Faculty of Physics, Astronomy and Applied Computer Science, Jagiellonian University, Krakow, Poland

**Keywords:** Aging, Physical activity, Capillarization, Angiogenesis, Lifestyle modification, Preventive medicine

## Abstract

Fast locomotory muscles, which are responsible for generating the highest power outputs, are more vulnerable to aging than slow muscles. In this study, we aimed to evaluate the impact of middle age and voluntary physical activity on capillarization and angiogenic potential in fast locomotory muscles. Middle-aged (M-group) and young (Y-group) wild-type FVB female mice were randomly assigned to either the sedentary or trained group undergoing 8-week spontaneous wheel running (8-sWR). Capillary density (assessed via immunohistochemical capillary staining and Western immunoblotting) of the fast locomotory muscles in the M-group (15-months old) was not significantly different compared to the Y-group (4-months old). Nevertheless, the expression of key pro-angiogenic genes in the fast muscle of the M-group was lower than that in the fast muscle of Y-group. 8-sWR had no impact on muscle capillarization; however, it increased fast muscle *Vegfa* expression in both the M and Y groups. We concluded that although fast muscle capillarization is still preserved in middle age, nevertheless the angiogenic potential (at least at the level of gene expression) is significantly reduced at this stage of aging. Moderate-intensity voluntary physical activity had no effect on capillary density, but it increased the angiogenic potential of the fast muscle.

**Supplementary Information:**

The online version contains supplementary material available at 10.1038/s41598-025-93176-1.

## Introduction

Skeletal muscle atrophy (sarcopenia) and a decline in muscle strength (dynapenia) accompany the physiological process of aging, leading to a decrease in physical exercise performance and negative health-related consequences in humans, such as fall-related injuries^1^. Sarcopenia in humans starts as early as the 3rd decade of life; however, a turning point in this process occurs in middle age (~ 50–60 years), when a noticeable decrease in skeletal muscle mass is observed, and furthermore, the loss of muscle mass accelerates^2,3^.

One of the important features of age-related sarcopenia is the loss of muscle capillaries, which are essential for adequate muscle blood perfusion, metabolic stability and exercise capacity^4–7^. In human skeletal muscles, each muscle fiber is surrounded by ~ 3–6 capillaries, and muscle capillary density differs depending on the muscle fiber type (which is greater in red, oxidative muscle fibers than in glycolytic muscle fibers) and training status^8^. Muscle microcirculation is responsible for both the delivery of nutrients (carbohydrates, fatty acids, and amino acids) and oxygen (for the mitochondrial oxidative phosphorylation process) as well as for the removal of the byproducts of energy metabolism (e.g., carbon dioxide, hydrogen ions, and lactate)^5–7^.

Substrates delivery may indeed be a key limiting factor of muscle tissue maintenance in aging^9^. However, the data concerning the impact of aging on muscle capillarization are inconsistent, since some studies reported no effect of aging on muscle capillarization^10–12^, whereas others showed a reduction in muscle capillary density^13,14^. Interestingly, it has been shown that aging primarily affects fast-twitch muscle fibers (with lower capillary density) that are recruited to generate high power outputs during exercise^15,16^. Hence, the ability to perform high-intensity exercise (especially at the intensity corresponding to the maximal power output) is impaired in aged people, in contrast to the ability to perform endurance activity, which is better preserved during aging^1,17^.

In accordance with the concept that aging compromises muscle capillarization, lowering muscle capillarization (as in advanced age)^18^ is associated with, among other factors, a decrease in satellite and endothelial cell function and content^19^ and an impairment of muscle metabolism, including disturbances in glucose metabolism^20^. As a consequence, the age-dependent decline in muscle capillary density not only lowers physical exercise performance^18,21^ but also reduces muscle tissue regeneration potential^19^ and muscle metabolism, e.g., disturbing whole-body glucose homeostasis^20^. Regarding the last point one should be aware that since skeletal muscle accounts for ~ 40% of body mass in young individuals^22^, muscle metabolism at rest, especially during exercise, has a crucial impact on whole-body metabolism.

Physical training has been found to influence muscle capillary density^23–25^. First, a more extensive muscle capillary network is present in endurance-trained athletes, who possess a greater percentage of mitochondria-rich, oxidative muscle fibers in their locomotory muscles than sprinters^23,26^. It has also been demonstrated that physical training in previously untrained individuals augments the number of capillaries in skeletal muscles, both in young^27,28^ and older subjects^27^. However, it should be emphasized that an enhancement of muscle capillarization following training in aged individuals is rather limited to subjects who have been practicing endurance training for many years, i.e., for more than two decades^14,29^. On the other hand, low-to-moderate intensity endurance training is a form of physical activity considered as non-pharmacological strategy for the prevention and treatment of several illnesses in humans^30,31^.

There is a general notion that a decrease in muscle capillarization and an attenuation of angiogenic potential in skeletal muscles are attributable mainly to advanced age (over the 6th – 7th percentiles of lifespan)^14^; however, in view of the evidence discussed above, there is a possibility that the age-related deterioration of the muscle capillary network begins at a much earlier stage of life^32^. Therefore, the aim of the present study was to evaluate the impact of middle age (mice aged 15-months, which is ~ 40% of their lifespan expectancy) on capillarization (capillary density and capillary-to-fiber ratio) and angiogenic potential (the expression of key pro-angiogenic genes) in fast-twitch muscles since fast skeletal muscles are predominantly affected by aging. Furthermore, we aimed to determine the impact of 8-weeks of spontaneous wheel running (8-sWR) (which corresponds to moderate-intensity physical activity in humans) on capillary density and angiogenic potential in fast-twitch skeletal muscles. We hypothesized that (i) the early stage of aging is accompanied by a decrease in muscle capillarization and muscle angiogenic potential and that (ii) moderate-intensity spontaneous physical activity attenuates middle-age-induced harmful effects on the muscle capillary network.

## Results

### Physical performance of young and middle-aged mice

**Fig. 1:**
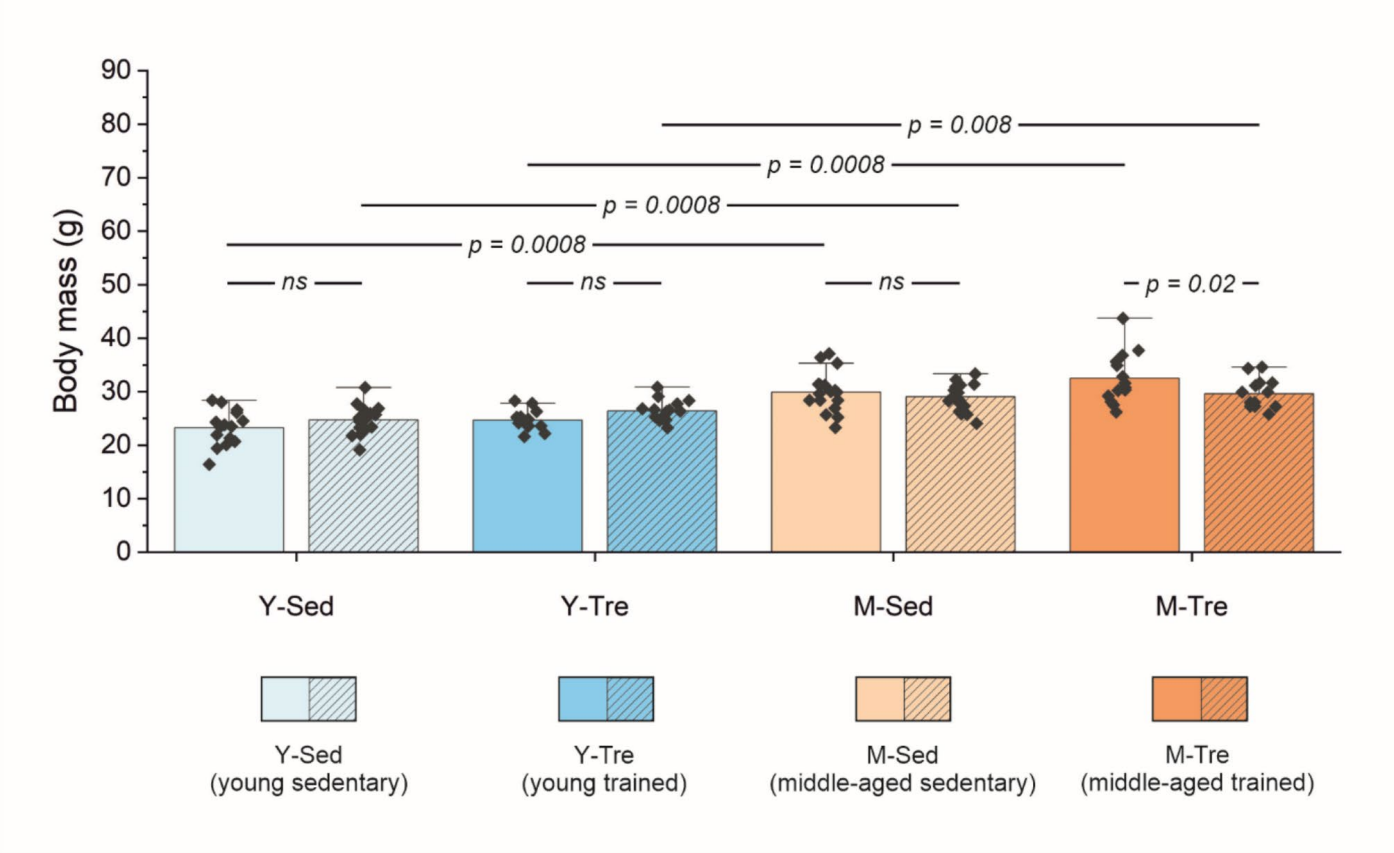
**Body mass before and after 8 weeks of the experiment in the groups of young (Y) and middle-aged (M) mice.** The data are presented as the means + SDs. Each data point in the dot plot represents one individual mouse. Two-way mixed ANOVA with one factor of repeated measure was used. Statistically significant changes (*p* < 0.05) are plotted on the graphs; *ns*, not statistically significant. The effect of age is shown by different colors of bars, i.e., blue for young and orange for middle-aged mice (with different shades for sedentary and trained groups). The effect of 8 weeks of the experiment (in the sedentary and trained groups) is marked by a striped pattern of bars. The number of mice in each group was as follows: Y-Sed (*n* = 15), Y-Tre (*n* = 15), M-Sed (*n* = 17), and M-Tre (*n* = 14).

The body mass of the middle-aged sedentary group of mice (M-Sed) at the start of the study was significantly greater (by ~ 29%) than that of the young sedentary group (Y-Sed) and remained greater (by ~ 18%) during the subsequent measurement performed after 8 weeks (Fig. 1). Eight weeks of spontaneous wheel running physical activity decreased the body mass (by ~ 9%) solely in the middle-aged trained group of mice (M-Tre), whereas the body mass was unaffected by 8-sWR in the young trained group of mice (Y-Tre) (Fig. 1).

**Fig. 2:**
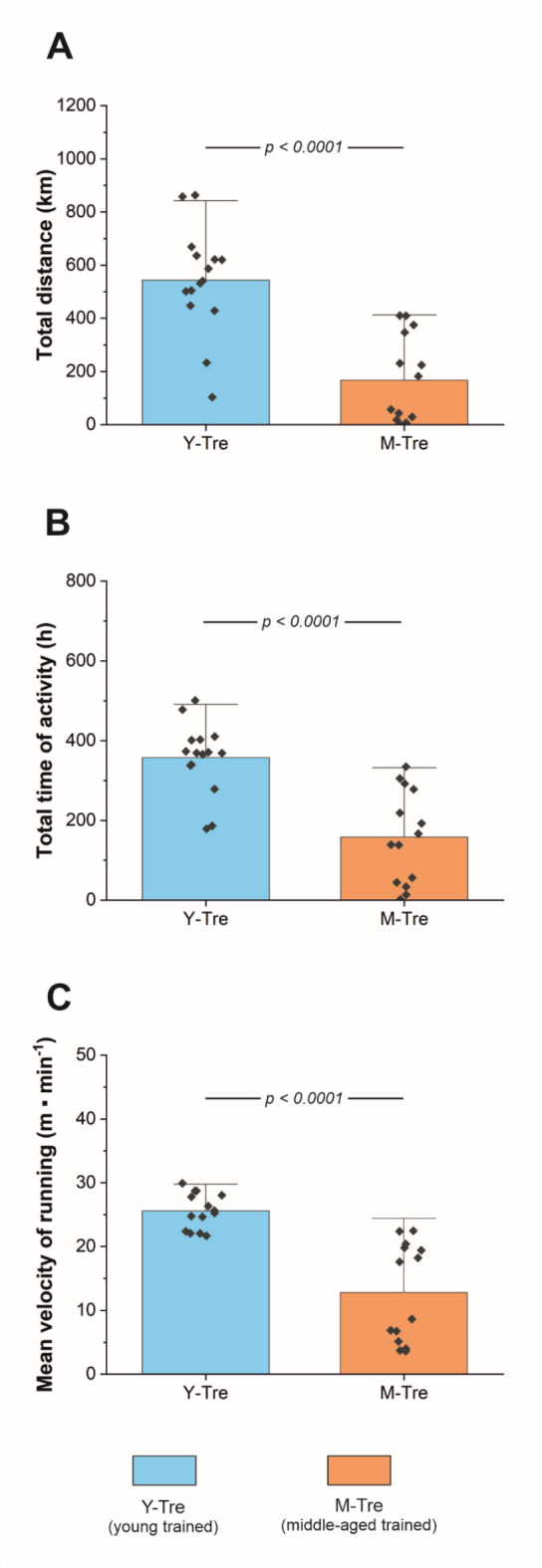
Physical performance of young (Y) and middle-aged (M) mice. Total distance covered during 8 weeks of spontaneous wheel running (8-sWR) (*n* = 15–14) (**A**); total time of activity during 8-sWR (*n* = 15–14) (**B**); and mean velocity during 8-sWR (*n* = 14–14) (**C**) in Y and M mice. The data are presented as the means + SDs. Each data point in the dot plot represents one individual mouse. Two-sided *p* values are shown (Mann–Whitney U test). Statistically significant changes (*p* < 0.05) were plotted on the graphs.

The exercise performance of the mice (recorded throughout 8-weeks of spontaneous wheel running) is presented in Fig. 2. We found that the total distance covered by the M-Tre group was ~ 69% lower than that covered by the Y-Tre group (167 ± 164 and 543 ± 199 km, respectively, for M-Tre and Y-Tre) (Fig. 2A). Moreover, the total time of activity in the M-Tre group was significantly lower (by ~ 56%) than that in their younger counterparts (158 ± 116 and 358 ± 89 h, respectively, for M-Tre and Y-Tre) (Fig. 2B). The mean velocity of running during 8-sWR was also lower (by ~ 50%) in the M-Tre group (Fig. 2C). However, interestingly, the maximal velocity of running recorded over the whole period of the experiment was greater (by ~ 6%, *p* = 0.027) in M-Tre mice than in Y-Tre mice (77.7 ± 2.9 and 73.6 ± 5.1 m min^− 1^, respectively, for M-Tre and Y-Tre).

In terms of voluntary activity per day, the distance covered per day by the middle-aged mice was 2.98 ± 2.93 km, whereas in their young counterparts, it was 9.70 ± 3.56 km. Similarly, the time spent in wheels per day in the M-Tre group of mice was 2.83 ± 2.07 h, which was lower (*p* < 0.0001) than that in the Y-Tre group of mice (6.38 ± 1.59 h).

The exercise performance of the mice in the trained groups (M-Tre and Y-Tre, *n* = 29) was negatively correlated (*p* < 0.05) with body mass before training. Specifically, we found a significant negative correlation between body mass and total distance (*r* = -0.53; *p* = 0.002), total time spent in wheels (*r* = -0.50; *p* = 0.005) and mean running velocity (*r* = -0.53; *p* = 0.003).

### Locomotory muscles capillarization after 8-weeks of spontaneous wheel running in young and middle-aged mice

**Fig. 3:**
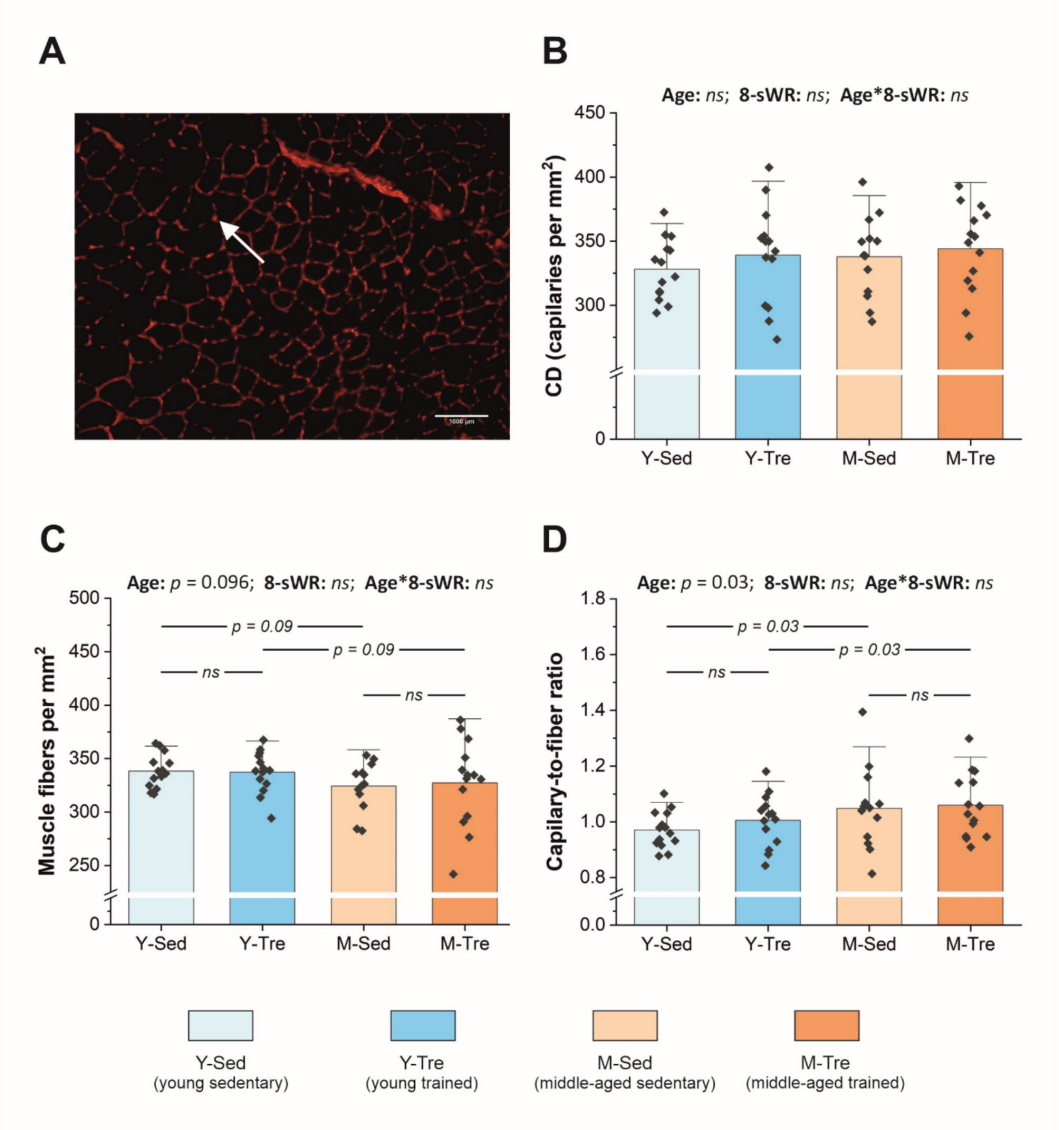
Muscle capillarization in the fast-twitch tibialis anterior (TA) muscle in the sedentary and trained groups of young (Y) and middle-aged (M) mice. Representative image of immunohistochemical staining with a platelet endothelial cell adhesion molecule antibody (anti-CD31, ab28364) at 20× magnification showing capillaries (red dot between muscle fibers indicated by white arrow) and muscle fibers (black fields) (**A**); the number of capillaries per mm^2^ of muscle cross-sectional area, i.e., capillary density (CD) (*n* = 14–14–13–14) (**B**); the number of muscle fibers per mm^2^ of muscle cross-sectional area (*n* = 14–14–13–14) (**C**); capillary-to-fiber ratio (*n* = 14–14–13–14) (**D**) in the fast-twitch TA muscle. The data are presented as the means + SDs. Each data point in the dot plot represents the mean measured value for one individual mouse. Two-way ANOVA followed by Tukey’s *post hoc* test was used. Statistically significant changes (*p* < 0.05) were plotted on the graphs.

In the present study, we assessed the muscle capillary density in the fast-twitch glycolytic tibialis anterior (TA) muscle on the basis of immunohistochemical staining of muscle cryosections for platelet endothelial cell adhesion molecule (CD31) (see representative image, Fig. 3A). We found no significant difference in muscle capillary density between middle-aged mice and their younger counterparts in the fast-twitch TA (Fig. 3B). Moreover, in the TA muscle, we observed a tendency toward a lower number of muscle fibers per mm^2^ of cross-sectional area in middle-aged mice than in young individuals in both the sedentary and trained groups (by ~ 3–4%, *p* = 0.09; Fig. 3C), whereas fiber cross-sectional area (FCSA) was not significantly different between the Y and M groups (Supplemental Fig. S1). Interestingly, the capillary-to-fiber ratio in the TA muscle was significantly greater (*p* = 0.03) in middle-aged mice than in their young counterparts in both the sedentary (by ~ 8%) and trained groups (by ~ 5%) (Fig. 3D).

**Fig. 4:**
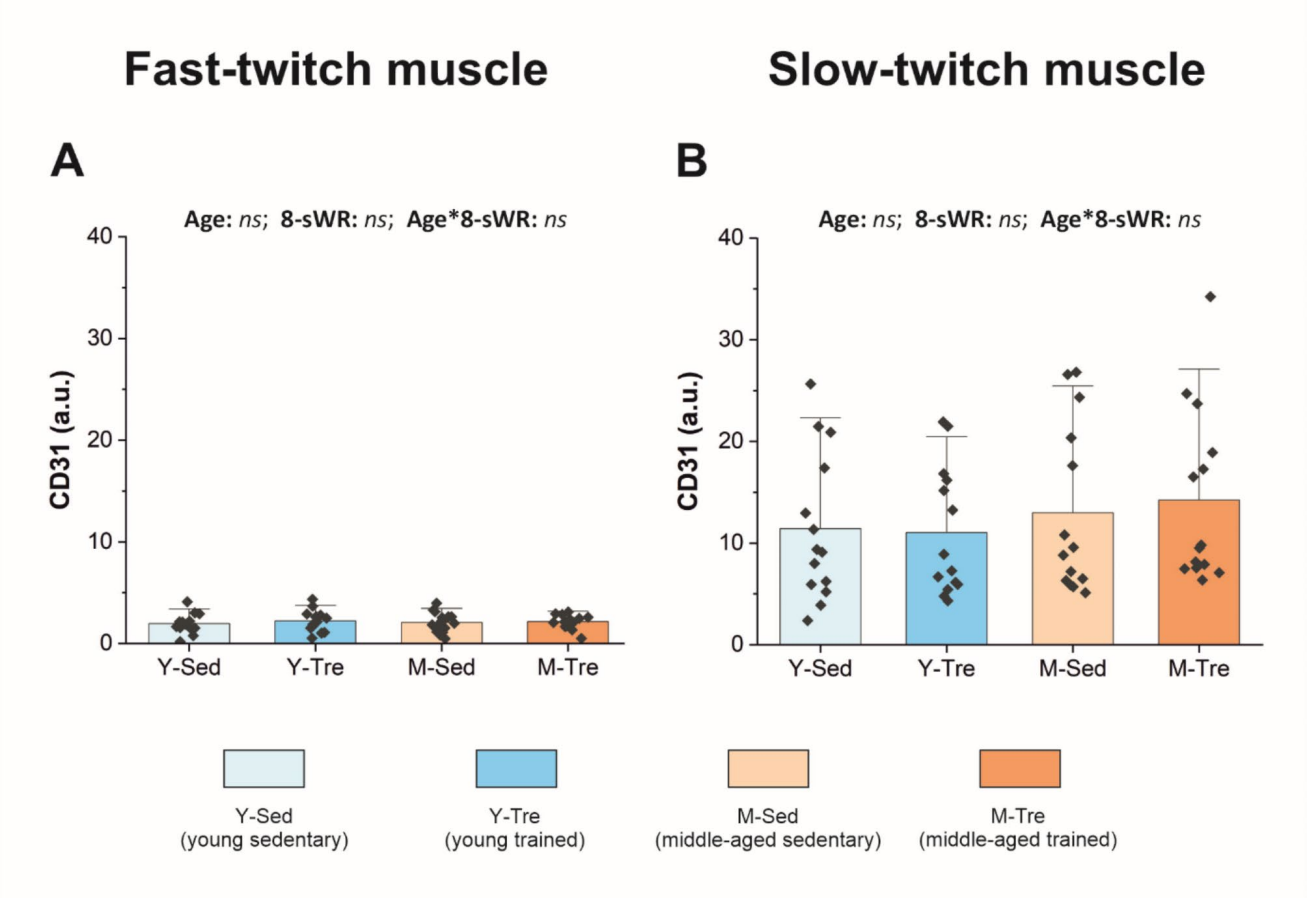
Platelet endothelial cell adhesion molecule (CD31) protein expression in the fast-twitch tibialis anterior (TA) and slow-twitch soleus (Sol) muscles in the sedentary and trained groups of young (Y) and middle-aged (M) mice. The content of CD31 (*n* = 14–15–17–14) in the TA (**A**) and Sol muscles (*n* = 14–14–14–14) (**B**). The data are presented as the means + SDs. Each data point in the dot plot represents one individual mouse sample. Two-way ANOVA followed by Tukey’s *post hoc* test was used. *ns*, not statistically significant. The representative blots and corresponding Ponceau detection results are shown in Supplemental Figure S6.

Owing to the shortage of muscle samples, we did not perform immunohistochemical analysis of the slow-twitch oxidative soleus muscle (Sol). However, we analyzed capillarization in both types of the locomotory muscles, i.e., the fast-twitch TA and slow-twitch Sol (CD31 protein expression), via Western immunoblotting (WB) analysis. We detected ~ 6-times lower levels of the CD31 protein in the fast-twitch TA muscle than in the slow-twitch Sol muscle under basal conditions (sedentary groups of young and middle-aged mice (Fig. 4A–B). However, there was no effect of middle age on CD31 protein expression in either the fast-twitch TA (Fig. 4A) or the slow-twitch Sol (Fig. 4B).

Eight weeks of spontaneous wheel running had no effect on capillary density (Fig. 3B), the number of muscle fibers per mm^2^ (Fig. 3C) or the capillary-to-fiber ratio (Fig. 3D) in the fast-twitch TA muscle in either young or middle-aged mice. Moreover, spontaneous wheel running had no effect on the CD31 protein expression in either the fast-twitch TA or the slow-twitch Sol (Fig. 4A–B). Interestingly, we found a similar increase (by ~ 7% and ~ 13% in the Y-group and M-group, respectively, no significant interaction) in the FCSA after the 8-sWR (Supplemental Fig. S1A).

### Angiogenesis-related gene expression in the fast-twitch muscle of young and middle-aged mice after 8 weeks of spontaneous wheel running

**Fig. 5:**
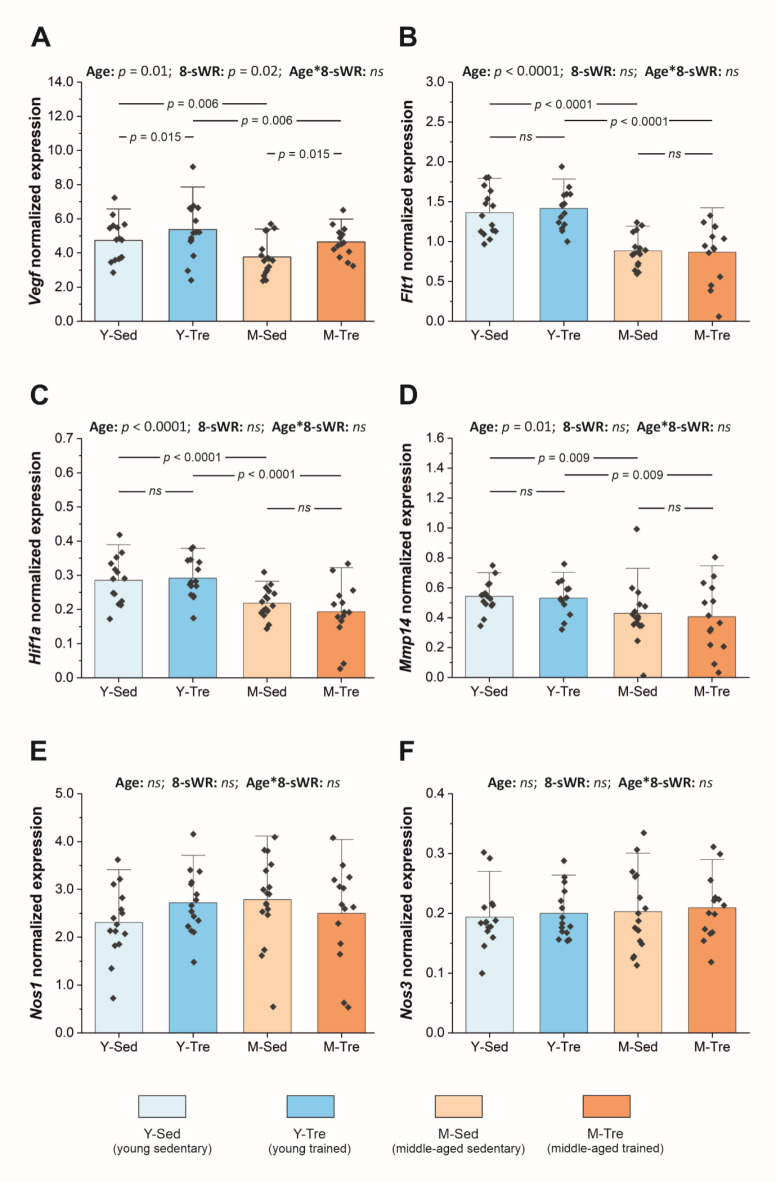
Pro-angiogenic genes expression in the fast-twitch extensor digitorum longus (EDL) muscle (normalized to a geometric mean of four reference genes) in the sedentary and trained groups of young (Y) and middle-aged (M) mice. The expression of vascular endothelial growth factor A (*Vegfa*) (*n* = 15–15–17–14) (**A**); FMS-like tyrosine kinase 1 (*Flt1*) (*n* = 15–15–16–14) (**B**); hypoxia-inducible factor 1, alpha subunit (*Hif1a*) (*n* = 14–15–17–14) (**C**); matrix metallopeptidase 14 (*Mmp14*) (*n* = 15–14–16–14) (**D**); nitric oxide synthase 1 (*Nos1*) (*n* = 15–15–17–14) (**E**); nitric oxide synthase 3 (*Nos3*) (*n* = 15–15–17–14) (**F**). The data are presented as the means + SDs. Each data point in the dot plot represents one individual mouse sample. Two-way ANOVA followed by Tukey’s *post hoc* test was used. Statistically significant changes (*p* < 0.05) are plotted on the graphs; *ns*, not statistically significant.

Compared with their young counterparts, middle-aged mice are characterized by significantly lower expression of pro-angiogenic genes, including vascular endothelial growth factor A (*Vegfa*) (by ~ 21%), FMS-like tyrosine kinase 1 (*Flt1*) (by ~ 35%), hypoxia-inducible factor 1, alpha subunit (*Hif1a*) (by ~ 24%) and matrix metallopeptidase 14 (*Mmp14*) (by ~ 20%), in the fast-twitch extensor digitorum longus (EDL) muscle (Fig. 5A–D). No effect of middle age (*p* > 0.05) on the expression of neuronal or endothelial nitric oxide (NO) synthases (*Nos1* and *Nos3*, respectively) in the fast-twitch EDL muscle was observed (Fig. 5E–F). Moreover, no significant difference (*p* > 0.05) in the expression of *Nos2* (the inducible isoform of NOS) was detected (data not presented).

Eight weeks of spontaneous wheel running significantly increased *Vegfa* expression in the fast-twitch EDL muscle in middle-aged (by ~ 23%) and young mice (by ~ 13%) (Fig. 5A), whereas physical activity had no effect of on *Flt1*, *Hif1a*, or *Mmp14* gene expression (*p* > 0.05, Fig. 5B–D). Additionally, *Nos1* and *Nos3* expression in the fast-twitch muscles of young and middle-aged mice was unaffected by physical training (*p* > 0.05, Fig. 5E–F). The expression of *Nos2* was significantly greater after 8-sWR, but only in the young group of mice (by ~ 38%, *p* = 0.0008; data not presented in the figure).

Figure 5 shows the results of the aforementioned pro-angiogenic genes expression in the fast-twitch EDL muscle (*Vegfa*, *Flt1*, *Hif1a*,* Mmp14*,* Nos1*,* Nos2*, and *Nos3*) normalized to the geometric mean of four reference genes, i.e., hypoxanthine guanine phosphoribosyl transferase (*Hprt*), hydroxymethylbilane synthase (*Hmbs*), ribosomal protein, large, P0 (*Rplp0*), and beta-actin (*Actb*) (Supplemental Fig. S2E). The mRNA expression of the reference genes in the EDL muscle (raw data) is presented in Supplemental Fig. S2A–D. Additionally, we present the expression of angiogenesis-related genes without normalization (Supplemental Fig. S3) and after normalization to *Hprt* gene expression (Supplemental Fig. S4). *Hprt* is a commonly used reference gene for normalization of genes of interest in skeletal muscles.

### Muscle oxidative metabolism and muscle antioxidant protection after 8 weeks of spontaneous wheel running in young and middle-aged mice

**Fig. 6:**
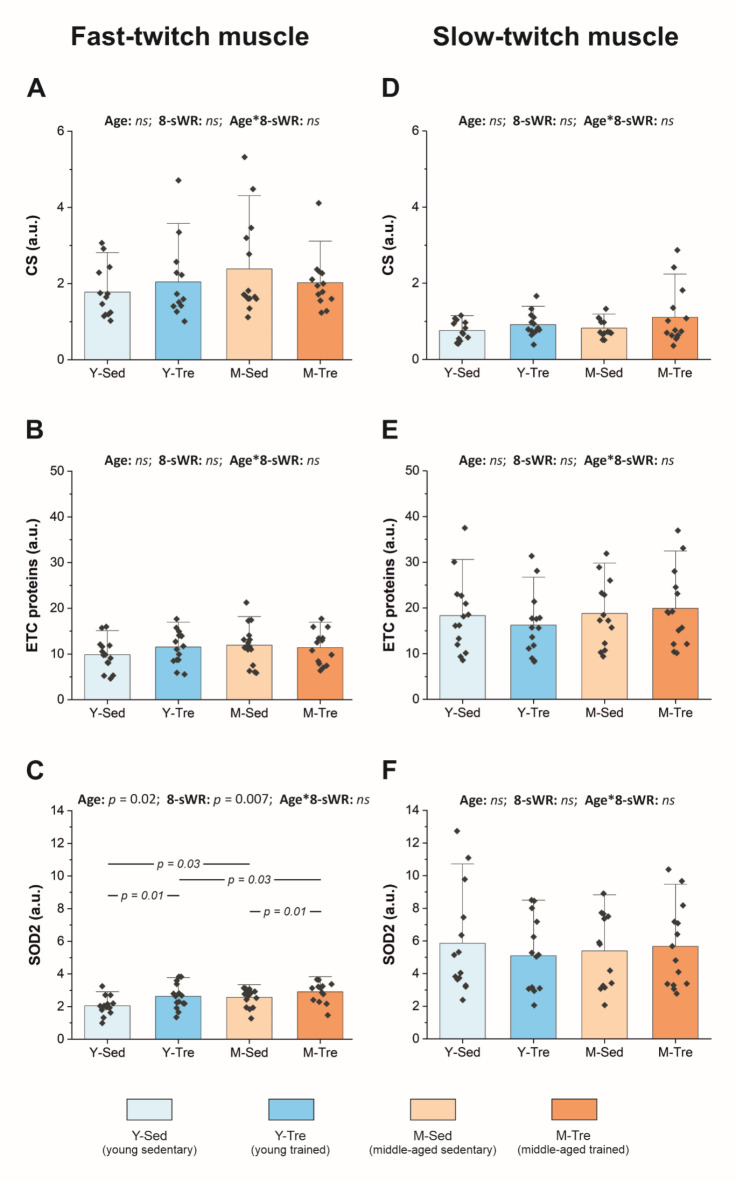
The contents of oxidative metabolism and antioxidant protection proteins in the fast-twitch tibialis anterior (TA) and slow-twitch soleus (Sol) muscles in the sedentary and trained groups of young (Y) and middle-aged (M) mice. The protein content of citrate synthase (CS) (*n* = 13–13–14–13) (**A**), the sum of electron transport chain (ETC) complexes (*n* = 14–15–17–14) (**B**) and superoxide dismutase 2 (SOD2) (*n* = 14–15–17–14) (**C**) in the TA muscle. The protein content of CS (*n* = 13–14–13–14) (**D**), the sum of ETC complexes (*n* = 14–14–13–14) (**E**) and SOD2 (*n* = 14–14–13–14) (**F**) in the Sol muscle. The data are presented as the means + SDs. Each data point in the dot plot represents one individual mouse sample. Two-way ANOVA followed by Tukey’s *post hoc* test was used. Statistically significant changes (*p* < 0.05) are plotted on the graphs; *ns*, not statistically significant. The representative blots and corresponding Ponceau detection in the fast-twitch TA and slow-twitch Sol are shown in Supplemental Figures S7 and S8, respectively.

In the present study, we used the amount of mitochondrial proteins of the electron transport chain (ETC) complexes, i.e., complex II (CII), complex III (CIII), complex IV (CIV) and complex V (CV), as proxies for mitochondrial content. Both the total amount of the analyzed mitochondrial ETC proteins (the sum of CII, CIII, CIV and CV) and the content of citrate synthase (CS), the key enzyme of the tricarboxylic acid cycle (TCA), were used as markers of muscle oxidative metabolism. Accordingly, we found no effect of middle age on the CS content (Fig. 6A) or on the sum of ETC protein complexes in the fast-twitch TA muscle (Fig. 6B). However, when the impact of middle age on mitochondrial antioxidant protection was considered, we detected significantly greater (by ~ 25%) superoxide dismutase (SOD2) protein expression in middle-aged mice than in their younger counterparts (Fig. 6C). The analysis of muscle protein content (CS, the sum of ETC protein complexes, and SOD2) in the slow-twitch Sol revealed no effect of middle age on protein expression (Fig. 6D–F).

Eight weeks of spontaneous wheel running had no effect on the oxidative metabolism markers in either the fast-twitch TA (Fig. 6A–B) or the slow-twitch Sol (Fig. 6D–E). Interestingly, 8-sWR increased SOD2 expression, both in young (by ~ 28%) and middle-aged mice (by ~ 13%), but only in the fast-twitch TA muscle (Fig. 6C).

In the Supplemental Materials, we also present the impact of aging and 8-sWR on the content of ETC complexes, i.e., CII, CIII, CIV and CV, both in the fast-twitch TA and in the slow-twitch Sol (Supplemental Fig. S5).

Additionally, we reported that the content of the sum of ETC proteins was ~ 2 times lower in the TA muscle than in the Sol muscle, both in young and middle-aged mice (Fig. 6B and E). Moreover, we found that the SOD2 protein content was lower in TA than in Sol in both young (~ 3 times) and middle-aged mice (~ 2 times) (Fig. 6C and F). These results confirm that oxidative muscles (such as the Sol) possess greater contents of mitochondria than do glycolytic muscles (such as the TA).

## Discussion

The main findings of the present study can be summarized as follows: (i) middle-aged (15 month-old, M-group) mice are characterized by reduced physical exercise capacity compared with young (4 month-old, Y-group) mice and tend toward fast muscle fiber atrophy; (ii) muscle capillarization in middle-aged mice is preserved, although a clear decrease in fast muscle angiogenic potential is present, as reflected by an attenuation of the expression of key pro-angiogenic genes; (iii) spontaneous wheel running lasting 8 weeks in both groups of studied mice (M and Y) had no impact on muscle capillary density, but it increased *Vegfa* mRNA expression in the fast locomotory muscle.

## Muscle capillarization at middle age

In the present study we demonstrated that exercise performance in middle-aged mice is markedly reduced, as reflected by a loss of daily and total (after 8 weeks of recording) exercise physical activity (Fig. 2). Accordingly, a significantly lower total distance covered (by ~ 69%), total time of activity (by ~ 56%) and mean running velocity (by ~ 50%) were found in middle-aged mice during 8 weeks of spontaneous wheel running than in their younger counterparts (Fig. 2A–C). The loss of voluntary exercise performance, present in middle-aged mice, suggests that the process of aging in mice begins far earlier before they reach advanced age (life expectancy reaching a maximum of 39 months of age)^33^, which is in accordance with earlier reports^34,35^. The age-dependent loss of exercise performance may be related to several processes that compromise the vital mechanisms responsible for animal locomotion, e.g., oxygen delivery to locomotory muscles and muscle energy system function^36^.

When considering muscle energy supply in this type of exercise, as applied in our study i.e., moderate-intensity prolonged voluntary wheel running, the main energy supply system is oxidative phosphorylation (OXPHOS), which depends on both oxygen delivery and oxygen utilization in mitochondria^6,37,38^.

Regarding oxygen delivery to the locomotory muscles, the age-dependent decline in the muscle capillary network has been found to reduce physical exercise performance^18,21^. Moreover, the results of recent studies show that muscle capillarization may be indeed a critical factor in the regulation of skeletal muscle mass maintenance and muscle functioning in aging^9^.

Interestingly, fast-twitch locomotory muscles are especially vulnerable to the effects of aging; i.e., aging affects fast-twitch muscles earlier than slow-twitch muscles^15^. The question arises, how the loss of muscle mass (especially the loss of fast-twitch muscle fibers) during aging, which accelerates at the ~ 6th decade of life in humans^2^, is related to muscle capillary rarefaction.

Accordingly, in the present study, we examined the effects of aging and moderate-intensity exercise on fast muscle capillary density in mice (Fig. 3). Fast muscle capillarization in our study was assessed through the analysis of the expression of CD31, a highly specific marker of vascular endothelial cells, via both immunohistochemistry (Fig. 3) and WB analysis (Fig. 4A). We found that fast muscle capillarization is well preserved in middle-aged mice, since no difference in capillary density between middle-aged mice and their young counterparts was observed (~ 338 vs. 328 capillaries per mm^2^ in M and Y mice, respectively) (Fig. 3B). This result is in agreement with the reports of other authors^32,39^. Unfortunately, owing to limited muscle tissue availability, we did not perform immunohistochemical capillary staining in the slow-twitch soleus. However, we carried out WB analysis of CD31 expression in this muscle (similar to that in fast-twitch TA) and found no effect of middle age on CD31 expression (Fig. 4B). Additionally, we confirmed that capillarization of the fast-twitch muscle was lower than that of the slow-twitch muscle^32^ in both groups of mice (by ~ 6 times, Fig. 4).

Despite the lack of impact of middle age on muscle capillary density, we found a tendency toward a lower number of muscle fibers in the fast-twitch TA (Fig. 3C). As a consequence, a higher capillary-to-fiber ratio in the TA of middle-aged mice than those of their young counterparts was observed (~ 1.05 vs. 0.97 capillaries per fiber in the M and Y groups, respectively; Fig. 3D). Interestingly, the fiber cross-sectional area did not differ between the M and Y groups (Supplemental Fig. S1A). This result shows that a decrease in the number of muscle fibers already manifests at middle age, whereas muscle capillarization at this stage of aging is still well maintained. This finding is in agreement with other authors’ suggestions^9^ that at middle age, muscle fiber atrophy precedes capillary rarefaction.

## Muscle angiogenic potential at middle age

In the present study, despite the lack of effect of middle age on fast muscle capillary density (Figs. 3 and 4A), we detected a significant decrease in the expression of key angiogenesis-related genes. Specifically, we detected reduced expression of *Vegfa* (by ~ 21%), *Flt1* (by ~ 35%) and *Hif1a* (by ~ 24%) in the fast-twitch muscle of middle-aged mice compared with young mice (Fig. 5A–C). In our study, the genes of interest, i.e., angiogenesis-related genes, were normalized to a geometric mean of the expression of the four reference genes, i.e., *Hprt*, *Hmbs*, *Rplp0* and *Actb* (Fig. 5). Before normalization, we checked the expression of the aforementioned reference genes (commonly used as reference genes in skeletal muscle gene expression analysis) and found that their mRNA expression in the fast-twitch EDL muscle was rather stable irrespective of age and 8-sWR (Supplemental Fig. S2). Additionally, since the *Hprt* gene alone is widely used as a reference gene in skeletal muscle, we also presented the data of our genes of interest normalized to *Hprt* mRNA expression (Supplemental Fig. S4). Both normalizations (to the geometric mean and to *Hprt* expression) yielded comparable results regarding the impact of middle age and 8-sWR on angiogenesis-related gene expression in the fast-twitch muscle (Fig. 5 and Supplemental Fig. S4).

Accordingly, we detected a decrease in the expression of crucial regulators of angiogenesis, such as *Vegf* and its receptor *Flt1*, which are responsible for the proliferation and migration of endothelial cells^40,41^. The key role of VEGF in the preservation of muscle capillarity was confirmed in a previous study, which demonstrated that *Vegf* gene inactivation leads to an ~ 64% reduction in capillarization in the gastrocnemius muscle of mice^42^. In this context, our results are in agreement with previous findings, showing that aging reduces *Vegf*,* Flt1* and *Hif1a* gene expression in the gastrocnemius muscle of mice^43^. As presented earlier, the expression of muscle VEGF is dependent on the expression of, e.g., the transcription factor HIF-1α^44^ and nitric oxide (NO) availability, which stabilize HIF-1α and upregulate VEGF production^45^. Our results revealed a decrease in *Hif1a* expression in the fast-twitch muscle at middle age (Fig. 5C), suggesting that attenuation of *Vegfa* expression in middle-aged mice may be a consequence of decreased HIF1α. On the other hand, the levels of the neuronal and endothelial isoforms of NO synthase were unaffected by aging (Fig. 5E–F). Therefore, the cause–effect relationships among *Vegf*,* Hif1a* and *Nos* at the level of gene expression need further study. Moreover, given that muscle satellite cells are closely spatially related to endothelial cells and that *Vegfa* is highly expressed in both active and quiescent muscle satellite cells^46^, our results suggest that in middle-aged individuals, an impairment in VEGF-mediated signaling may contribute not only to a decrease in muscle angiogenic potential but also to an attenuation of muscle regenerative capacity^19^. Indeed, it has been demonstrated that in advanced age, the regenerative capacity of muscle is severely reduced^19,47^. Although, the involvement of satellite cells in the etiology of age-related sarcopenia is debated, aging affects the regenerative capacity of skeletal muscle by reducing the size of the satellite cell pool and its functionality^48,49^.

Additionally, we found (at the gene expression level only) that middle age influences the extracellular matrix composition, since it decreases muscle matrix metallopeptidase 14 expression (*Mmp14*), which is involved in the remodeling and degradation of the basement membrane (necessary stage of building new vessels) (Fig. 5D). In summary, on the basis of our results, fast muscle capillary density and consequently, oxygen delivery to locomotory muscles seem to be unaffected at middle age, but one must be aware that a decrease in the angiogenic and regenerative capacity of fast skeletal muscle (at least at the level of gene expression) is already present at this stage of aging.

### Muscle oxidative metabolism in middle-aged individuals

As mentioned above, the attenuation of physical performance in middle-aged mice (Fig. 2) may be related to impaired the muscle energy resynthesis, especially in OXPHOS. Interestingly, it has been shown that aging affects oxidative metabolism differently in various muscles^50,51^. Specifically, it has been demonstrated that red, oxidative muscles (such as the soleus) maintain better mitochondrial function throughout aging than do glycolytic muscles (such as the tibialis anterior or gastrocnemius)^50,51^.

In the present study, we confirmed earlier results that glycolytic muscles (such as the TA) possess a lower content of mitochondria than do oxidative muscles (such as the soleus)^52^. When the impact of middle age on muscle oxidative metabolism was considered, we found that the contents of mitochondrial oxidative metabolism proteins (TCA and OXPHOS) in both the fast-twitch TA (Fig. 6A–B) and the slow-twitch Sol (Fig. 6D–E) of middle-aged mice were maintained at levels comparable to those of young animals. However, the possibility of age-related attenuation of mitochondrial oxidative metabolism protein activity should be taken into consideration^50,53,54^. It has been argued that the activity of the mitochondrial respiratory complex IV, which is the best measure of muscle capacity to synthesize ATP in OXPHOS^55^, is attenuated in aging, as it has indeed been shown that complex IV activity decreases with age^56,57^.

Notably, one of the causes of the greater vulnerability of fast-twitch muscles to aging (compared with slow-twitch muscles) is the greater production of reactive oxygen species by fast-type muscle fibers^58^. The significantly elevated expression of SOD2 in fast-twitch glycolytic TA muscle in the middle-aged mice in our study (Fig. 6C), in view of the lack of age-dependent changes in the SOD2 content in the slow-twitch oxidative soleus (Fig. 6F), indeed indicates that fast muscles may be especially subjected to age-related oxidative stress, hence exhibit compensatory adaptations^59,60^. Accordingly, as mentioned above, we found a tendency toward a lower number of muscle fibers in the fast-twitch TA muscle of middle-aged mice than in their younger counterparts revealing the potential effect of age-related oxidative stress on fast muscle atrophy (Fig. 3C). However, on the other hand, the fiber cross-sectional area in the TA muscle has been found to be well maintained at middle age (Supplemental Fig. S1A). This finding suggests that in fast muscles, the loss of muscle fiber numbers during aging precedes a decrease in muscle fiber size.

### Physical activity and muscle capillarization in young and middle-aged mice

In the present study, we were interested in answering the question of whether moderate-intensity voluntary physical activity (e.g., spontaneous wheel running), which is recommended for prevention and treatment of a variety of non-communicable diseases^31^, may be effective in increasing oxygen delivery to locomotory muscles (through an augmentation of muscle capillarization) as well as in enhancing oxygen utilization in OXPHOS^61^.

Accordingly, we found no effect of moderate-intensity voluntary physical activity on fast muscle capillarization in either young or middle-aged mice (Fig. 3B and D). In contrast, some authors reported that, in young mice, short-term voluntary wheel running effectively improved fast muscle capillarization (by ~ 44% after 1 week and by ~ 78% after 6 weeks)^62,63^. On the other hand, in accordance with our data, Baum and coworkers^64^ recently demonstrated that even forced treadmill running in mice lasting 4 weeks (performed 6 times per week, exercise bouts lasting 45 min, treadmill speed from 16 m/min at the beginning of training up to 25 m/min, treadmill incline from 10^o^ to 20^o^) had no effect on fast-twitch TA muscle capillarization in young (4-month-old) mice. Nevertheless, on the basis of studies in humans, high-intensity physical training (80–100% VO_2max_) appears to be more effective in terms of increasing muscle capillarization than low-intensity training (< 50% VO_2max_)^24^. The aforementioned data suggest that training-induced changes in muscle capillarization require much stronger stimuli than those induced by our voluntary moderate-intensity training.

However, moderate-intensity training, as applied in the present study, affected the expression of some angiogenesis-related genes, as we demonstrated a significant increase in *Vegfa* expression (Fig. 5A) in both groups of studied mice (M and Y). Moreover, we observed no changes in the levels of other pro-angiogenic genes in the fast-twitch muscles after 8-sWR (Fig. 5B–F). This finding is in agreement with previous studies showing that moderate-intensity training, which lasts 8 weeks (~ 65% of VO_2max_), significantly increases (by ∼82%) *Vegf* gene expression in the skeletal muscle of young men^16^. Moreover, this result suggests that a key factor of muscle angiogenic potential (*Vegf*) is sensitive even to moderate-intensity voluntary physical activity. One should take into account the recently proposed role of *Vegfa* in the cooperation between endothelial and satellite cells in muscle^19,46^ as discussed above. Therefore, our results may indicate that moderate-intensity training not only improves the angiogenic potential of fast muscle, but also enhances the regenerative capacity of muscle.

Interestingly, we also detected that 8 weeks of wheel running resulted in an increase in the muscle fiber size (by ~ 7% and ~ 13% in the Y-group and M-group, respectively) (Supplemental Figure S1 A). It need to be noticed that the literature data regarding the impact of endurance physical training on the FCSA are inconclusive. For example no effect of 5 weeks of treadmill training on the fiber CSA in rats was demonstrated in the study by Degens et al.^65^. In humans, moderate-intensity home-based training lasting 6 months resulted in a non significant increase (by ~ 8%) in FCSA in vastus lateralis muscle^28^. The observed in the present study relatively small, but statistically significant increase in the FCSA of the TA muscle after spontaneous wheel running noticed both in young and middle-aged mice suggests that moderate-intensity endurance training in middle-aged humans despite of improving endurance performance may also act toward protection of muscle mass.

### Physical activity and muscle oxidative metabolism in young and middle-aged mice

Even moderate-intensity endurance activity for a short duration (weeks) increases the expression of ETC proteins^66^, including complex IV activity and content^55^, in trained animals and in human muscles^37^. Additionally, moderate-intensity endurance training effectively increases muscle oxidative metabolism in older individuals^54^.

However, in the present study, we found no impact of 8-sWR on the locomotory muscle oxidative metabolism markers (CS and ETC protein contents) in either the fast-twitch TAs (Fig. 6A–B and Supplemental Fig. S5A–D) or the slow-twitch Sols (Fig. 6D–E and Supplemental Fig. S5E–H) of young and middle-aged mice. Interestingly, we detected an increase in SOD2 expression after 8-sWR, solely in the fast-twitch TA muscle, in both young and middle-aged mice (Fig. 6C). This result may indicate that physical activity-related reactive oxygen species production leads to an increase in the levels of muscle antioxidant proteins, such as the key antioxidant protein^59^, the mitochondrial isoform of superoxide dismutase (SOD2)^67^.

Nevertheless, the results of our study show that moderate-intensity physical activity is not sufficient to improve oxidative metabolism, at least as judged by the protein content, in the fast and slow-twitch mice locomotory muscles.

## Conclusions

Taken together, our results show that although the capillary density in the fast locomotory muscles is preserved in middle-aged of the animals, there is already a reduced angiogenic potential of the fast muscles at this stage of aging. Furthermore, we found that moderate-intensity physical activity had no effect on fast muscle capillarization but increased muscle angiogenic and regenerative potential in the mice studied. Our study implies that moderate-intensity physical activity applied at middle age in humans may play an important role in preventing and/or slowing the age-related decrease in the angiogenic potential and regenerative capacity of muscle tissue.

### Limitations of the study

Notably, in the present study, increased pro-angiogenic potential after physical activity was found only at the level of pro-angiogenic gene expression. This finding illustrates that the observed dose of spontaneous physical activity was sufficient to stimulate pro-angiogenic response at the gene level; however, it was not sufficient to induce changes in muscle capillarization. Most likely, more intense and/or longer training regimes are needed to induce this effect. Therefore, further studies are required to explore the effects of more intense (forcible) exercise training protocols on muscle capillarization during aging, examined both at the protein and morphological levels.

## Methods

### Ethical approval

Experimental protocols involving animals were approved by the II Local Ethics Committee on Animal Experimentation in Krakow (Permit Number: 914/2012; 37/2013; 27/2014) and were in compliance with the guidelines of the European Community Council Directive 2010/63/EU of 22 September 2010 on the protection of animals used for scientific purposes. The study is reported in accordance with the ARRIVE guidelines.

### Animals

A total of sixty-two female wild-type FVB mice (Animal Laboratory of the Medical Research Centre of the Polish Academy of Sciences, Warsaw, Poland), i.e., thirty-one, 2-month-old young mice (Y) and thirty-one 13-month-old middle-aged mice (M), were included in the study. The mice were randomly assigned to sedentary (Sed) or training (Tre) groups. Each individual mouse was considered the experimental unit within this study. One mouse died during the experiment and was excluded from the initial group. The body mass and final sizes of the groups were as follows: young sedentary group (Y-Sed, *n* = 15): 23.23 ± 3.33 g of body mass; young trained group (Y-Tre, *n* = 15): 24.65 ± 1.82 g of body mass; middle-aged sedentary group (M-Sed, *n* = 17): 29.92 ± 3.78 g of body mass; and middle-aged trained group (M-Tre, *n* = 14): 32.53 ± 4.76 g of body mass.

During the experiment, the animals were kept in standard laboratory cages in a room with a 12 h/12 h light/dark cycle and controlled temperature (22–24 °C) and humidity (~ 55%). All the mice had unrestricted access to standard rodent chow (Altromin, 1320) and tap water. Experimental blinding and randomization strategies were employed when possible. The sample size between 14 and 17 in each group for a particular measurement was evaluated on the basis of the results obtained from our previous studies.

### Wheel running activity

Eight weeks of spontaneous wheel running (8-sWR) was performed as described previously^35^. The mice in the trained groups were housed individually in cages equipped with running wheels (9.2 cm in diameter), which allowed them to perform 8-sWR, whereas the mice in the sedentary groups did not have access to the running wheels. The distance covered during voluntary running and the mean running velocity were calculated on the basis of the number of revolutions of the wheel, its radius and the time of activity. The individual data of the covered distance and the mean running velocity were expressed as the mean + standard deviation (SD) value every 24 h and were further averaged for the entire period of training (8 weeks, i.e., 56 days). The total time of activity in running wheels of each mouse was calculated by dividing the total distance by the mean velocity of running and was expressed as the mean + SD. In the experiment, we analyzed the mean velocity of running for the whole experiment (*v*_mean_ equals the covered distance divided by the total time of activity in the wheels), and the maximum instantaneous velocity (*v*_max_) was considered the maximum instantaneous speed recorded over the whole time of the experiment.

### Muscle tissue dissection

At the end of the experiment (after 8 weeks), young (4 months old) and middle-aged (15 months old) mice from the trained groups (within 24 h after the last running activity) and from the sedentary groups were sacrificed by cervical dislocation. All efforts were made to minimize the suffering of the experimental animals. Locomotory muscles with various muscle fiber type composition, i.e., the fast-twitch glycolytic muscles, tibialis anterior (TA) and extensor digitorum longus (EDL), as well as the slow-twitch oxidative soleus (Sol) muscle, were dissected immediately after the animals were killed (between 5 and a maximum of 10 min after cervical dislocation) and frozen in liquid nitrogen (LN_2_). Tissue extraction from all the animals was conducted by the same person in the same sequence as follows: (i) Sol, (ii) TA, and (iii) EDL. Care was taken to dissect the samples in the abovementioned order at the same time after the animals were killed.

The fast-twitch muscles of mice, i.e., the TA and EDL, are characterized by a prevalence of fast-twitch glycolytic muscle fibers with low mitochondrial content (~ 94% and ~ 88% of the fast-twitch muscle fibers, respectively, for the TA and EDL)^68^. In contrast, Sol in mice contains up to ~ 11% fast-twitch glycolytic muscle fibers and ~ 89% mitochondria-rich, oxidative muscle fibers^69^.

### Protein extraction and western immunoblotting analysis

Protein extraction from the analyzed muscles (TA and Sol) was performed as described previously^52^. The protein concentration in the homogenates was measured via a NanoDrop 2000 UV Vis Spectrophotometer (Thermo Fisher Scientific™, Waltham, MA, USA). The muscle extracts were stored at − 80 °C until further analysis.

The muscle-derived protein extracts were separated via 4 to 20% gradient gels (Mini-PROTEAN TGX gels, Bio-Rad Laboratories, Hercules, CA, USA). Equal amounts of total protein were loaded on gels. To eliminate differences between the gels resulting from unequal transfer, an internal standard (i.e., a gastrocnemius muscle sample from an adult mouse) was applied to each gel. The proteins were then transferred onto nitrocellulose membranes (Amersham Hybond, GE Healthcare, Pittsburgh, PA, USA) at a constant voltage (35 V) in transfer buffer at 4 ºC. Following transfer, the protein bands on the membranes were detected via Ponceau S staining (0.1% w/v in 5% acetic acid, Merck KGaA, Darmstadt, Germany) to ensure equal loading and transfer of proteins. After Ponceau S staining, the membranes were incubated with primary antibodies (Abcam, Cambridge, UK) specific to the following: the platelet endothelial cell adhesion molecule (CD31) (ab28364); citrate synthase (CS) (ab129095); mitochondrial isoform of superoxide dismutase 2 (SOD2) (ADI-SOD-111, Enzo, Life Sciences, Farmingdale, NY, USA); and total OXPHOS rodent WB antibody cocktail (ab110413) containing 5 mouse monoclonal antibodies against ETC complexes: CI subunit NDUFB8 (ab110242), CII (ab14714), CIII-Core protein 2 (ab14745) CIV subunit I (ab14705) and CV alpha subunit (ab14748). After primary antibody incubation, the membranes were washed and incubated with secondary antibodies conjugated with horseradish peroxidase. The protein bands were visualized via an enhanced chemiluminescence method, and the data were imaged via GeneGnome 5 Syngene (GenSys 1.2.7.0, Syngene Bio Imaging, Cambridge, UK). Gene Tools Syngene analysis software was used for densitometric analysis. The optical density values obtained for proteins detected in the skeletal muscle samples were normalized to the internal standard and then to the protein content via Ponceau S staining^52^. The protein content is presented in arbitrary units.

### Immunohistochemistry

The frozen medial parts of the TA muscles were cut into 10-µm-thick cryosections at − 20 °C (CM 1850 UV, Leica, Nußloch, Germany), collected on microscope slides coated with polysine (Menzel Gläser, Thermo Fisher Scientific™, Waltham, MA, USA) and fixed in 4% paraformaldehyde in phosphate buffered saline (PBS). Blocking solution (10% horse serum in 0.1% Triton X-100-PBS) was applied for 1 h at RT. For the detection of capillaries, muscle cryosections were incubated overnight at 4 °C with an antibody specific for CD31 (dilution 1/100; ab28364; Abcam, Cambridge, UK). Next, the slides were washed in 0.1% Triton X-100-PBS and incubated with a goat anti-rabbit (Cy3) secondary antibody conjugated with fluorescent cyanine dye (dilution 1/2500; ab6939, Abcam, Cambridge, UK) for 1 h at RT. The slides were again washed in 0.1% Triton X-100-PBS and in PBS and subsequently fixed with 4′,6-diamidino-2-phenylindole (DAPI) (Merck KGaA, Darmstadt, Germany) via Fluoroshield.

Images of the fast-twitch TA muscle cryosections were visualized and captured at 20× magnification under a fluorescence microscope (Axio Scope, Zeiss, Oberkochen, Germany) via a Canon PowerShot G10. The number of images taken depended on the quality of the muscle cross-sections (between 6 and 24 images for each mouse). To assess fast-twitch muscle capillarization, a square lattice test system was used. Quantitative analyses were performed via ImageJ software (version 1.52a; National Institute of Health, Bethesda, Maryland, USA) by a single operator. The quantification of muscle capillarization was performed on the basis of capillary density (CD), i.e., the number of capillaries per mm^2^ of muscle, and the capillary-to-fiber ratio, i.e., the number of capillaries per muscle fiber. Moreover, the number of muscle fibers per mm^2^ of muscle was determined. Owing to the shortage of soleus muscle samples we could not perform this analysis in the slow-twitch muscle.

### Muscle fiber cross-sectional area measurement

The muscle fiber cross-sectional area (FCSA) was quantified manually via ImageJ software (version 1.52a; National Institute of Health, Bethesda, Maryland, USA). Individual fibers were identified in microscopy images of TA transverse sections on the basis of the intensity and localization of CD31-positive capillaries surrounding each muscle fiber. To delineate the fiber perimeter, we used the ImageJ polygon selection tool, which allows manual tracing of the edge of fibers and calculation of the individual fiber CSA with software programs. A grid of lines was applied to the image and the area of each square was 25 square inches. Six adjacent squares were defined as regions of interest (ROIs) and 5 individual fibers were outlined in each square and their FCSA was calculated. If the features of the fibers were difficult to distinguish in any square, another field was randomly selected. The analysis was performed independently by two people working in parallel on different sets of microscopy images (up to 5 microscopy images per person) from the same mouse. At the end of the analysis, the mean FCSA value for each mouse from the Y and M groups was calculated (~ 10 images × 5 fibers × 6 areas equal to 300 FCSA results). Finally, the data obtained in square inches were converted to square micrometers.

### RNA extraction, reverse transcription and real-time PCR

Frozen fast-twitch EDL muscle samples were homogenized in TRIzol reagent (Thermo Fisher Scientific™, Waltham, MA, USA), and total RNA was extracted according to the manufacturer’s protocol. The RNA concentration and quality were assessed via a Nano Drop 2000 spectrophotometer (Thermo Fisher Scientific™, Waltham, MA, USA). Reverse transcription was performed using Omniscript Reverse Transcriptase enzyme (Qiagen Inc., Valencia, CA, USA) utilizing 500 ng of total RNA isolated from EDL muscle at 37 °C for 60 min.

Real-time PCR was performed via Assay-On-Demand TaqMan probes, i.e., vascular endothelial growth factor A (*Vegfa*, Mm00437306_m1); FMS-like tyrosine kinase 1 (*Flt1*, Mm00438980_m1); hypoxia-inducible factor 1, alpha subunit (*Hif1a*, Mm00468869_m1); matrix metallopeptidase 14 (membrane-inserted) (*Mmp14*, Mm00485054_m1); nitric oxide synthase 1, neuronal (*Nos1*, Mm01208059_m1); nitric oxide synthase 2, inducible (*Nos2*, Mm00440502_m1); nitric oxide synthase 3, endothelial cell (*Nos3*, Mm00435217_m1), according to the manufacturer’s protocol (Applied Biosystems, Foster City, CA, USA), and was run on the CFX96 Real-Time system (Bio-Rad, Hercules, CA, USA). We used hypoxanthine guanine phosphoribosyl transferase (*Hprt*, Mm03024075_m1); hydroxymethylbilane synthase (*Hmbs*, Mm01143545_m1); ribosomal protein, large, P0, (*Rplp0*, Mm00725448_s1); and actin, beta (*Actb*, Mm02619580_g1) as reference genes. The abundance of RNA was calculated as 2^−(threshold cycle)^. We normalized the expression of genes of interest (angiogenesis-related genes, see above) using a geometric mean of four reference genes (*Hprt*, *Hmbs*, *Rplp0* and *Actb*)^70^. Additionally, we normalized the angiogenesis-related genes to the *Hprt* gene, the most frequently used reference gene in skeletal muscle RT–PCR analysis.

### Statistical analysis

The results of this study are presented as the means and standard deviations (SDs). The obtained data were examined for the presence of outliers via the Grubbs test. Statistical analyses were performed after the normality of distribution and homogeneity of variance were checked. For some variables (CS protein content in the TA muscle and CS, CD31, CIV, and CV protein contents in the Sol muscle), the original data were transformed to a logarithmic scale to perform valid analysis of variance.

Comparisons of the body masses of the mice in the different groups were performed via two-way mixed ANOVA (factors: age and 8-sWR), with one factor of repeated measurement (before and after 8 weeks). To compare the parameters of running performance (daily and total distance, daily and total time of activity as well as the mean velocity of running) of trained Y and M mice, the Mann–Whitney U test was performed and two-sided *p* values are shown. Moreover, Spearman’s rank correlation analysis was performed to study the relationships between body mass and running performance parameters. Two-way analysis of variance (ANOVA), followed by Tukey’s *post hoc* test, was used to evaluate the impact of age and spontaneous wheel running (8-sWR) on genes, protein expression and muscle capillarization variables. For *Rplp0* mRNA and *Mmp14* gene expression normalized to *Hprt* in EDL muscle, a Kruskal–Wallis test followed by Dunn’s *post hoc* test was performed. In the case of *Hmbs* mRNA gene expression in the EDL muscle, Welch ANOVA followed by a Games–Howell post-hoc test was used. Statistical significance was set at *p* = 0.05 and two-tailed *p* values are presented. To estimate the sensitivity of the experiment, some ANOVA power calculations were performed. For the balanced 2 × 2 ANOVA design with a total number of 61 observations, a significance level of *p* < 0.05 and standard effect sizes of f = 0.1, 0.25 and 0.40, which are conventionally attributed to small, medium, and large effects, the powers of the corresponding F tests were 0.12, 0.48, and 0.87 for the 2-level effects and for the interaction, respectively. *p* < 0.05 indicated statistical significance, and two-tailed *p*-values are presented. Statistical analyses of the data were carried out via Origin 2022 v 9.9 software (OriginLab Corporation, Northampton, MA, USA). All the statistical power calculations were computed via G*Power v.3.1.9.7.

## Electronic supplementary material

Below is the link to the electronic supplementary material.

Supplementary Material 1

## Data Availability

The datasets used and/or analyzed during the current study available from the corresponding author on reasonable request.
