# Supplementary material for: Physical activity reverses the aging induced decline in angiogenic potential in the fast locomotory muscles of mice
Source: Sci Rep. 2025 Mar 14;15:8848. doi: 10.1038/s41598-025-93176-1 (PMC11909166; doi:10.1038/s41598-025-93176-1)
Supplement: Supplementary file 1 — Supplementary Material 1 [file 41598_2025_93176_MOESM1_ESM.pdf]

Physical activity reverses the aging induced decline in angiogenic potential in the fast locomotory muscles of mice

Magdalena Zmudzka[1#], Joanna Szramel[1#], Janusz Karasinski[2], Zenon Nieckarz[3], Jerzy A. Zoladz[1], Joanna Majerczak[1]

Supplemental Figure S1

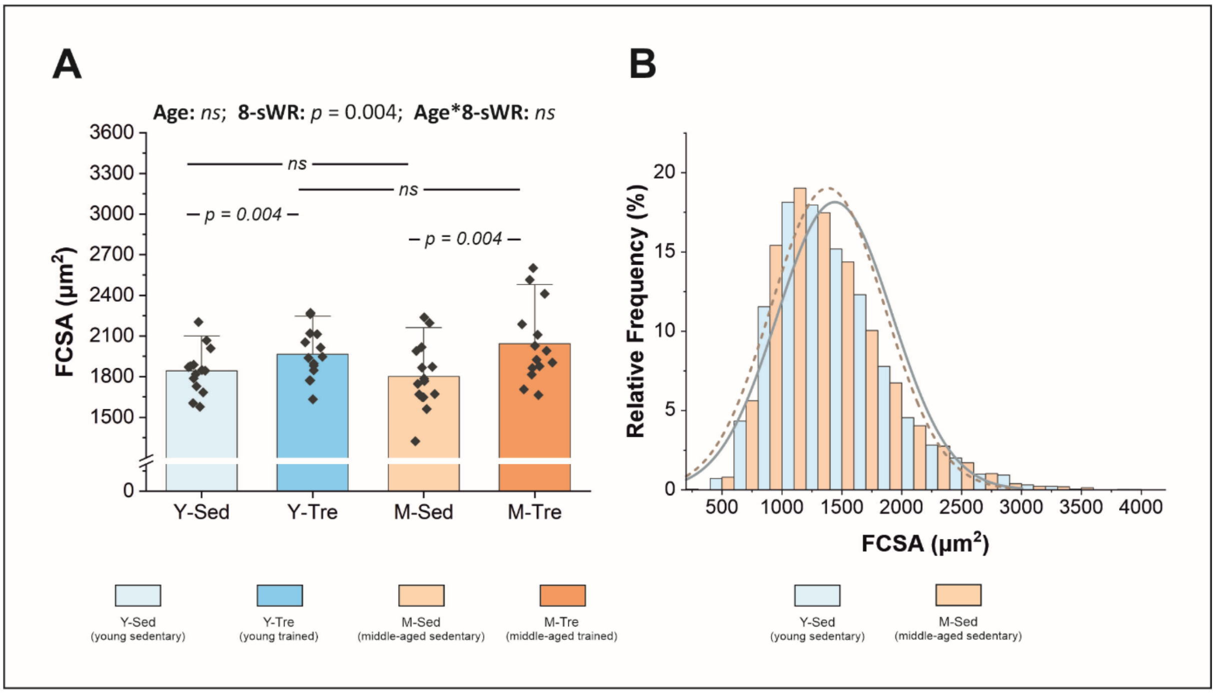

Supplemental Figure S1. Fiber cross-sectional area (FCSA) of the fast-twitch tibialis anterior (TA) muscle. FCSA in the TA muscle of the sedentary and trained groups of young (Y) and middle-aged (M) mice (n = 14–14–15–14) (**A**). The data are presented as the means + SDs. Each data point in the dot plot represents a mean value of the FCSA measured for each mouse**.** Two-way ANOVA followed by Tukey's *post hoc* test was used. Statistically significant changes ($p < 0.05$) are plotted on the graphs; *ns*, not statistically significant.
Distribution of the FCSA in the TA muscle of young sedentary and middle-aged sedentary groups of mice (on the basis of the 3186 vs 3632 FCSA results, respectively) (**B**).

Supplemental Figure S2

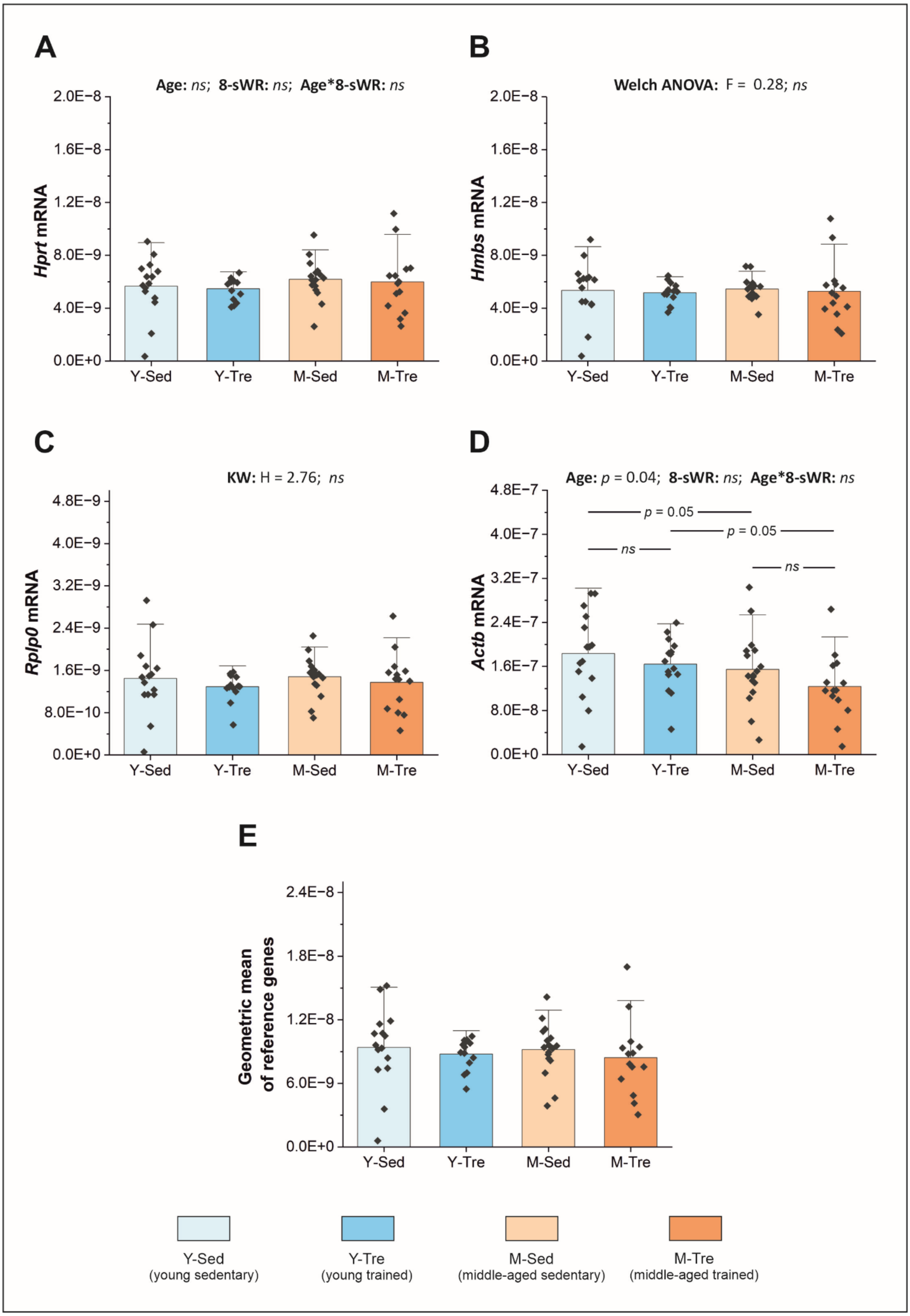

Supplemental Figure S2. Reference gene expression in the fast-twitch extensor digitorum longus (EDL) muscle in the sedentary and trained groups of young (Y) and middle-aged (M) mice. The mRNA expression of hypoxanthine guanine phosphoribosyl transferase (*Hprt*) (n = 15–14–17–14) (**A**); hydroxymethylbilane

synthase (*Hmbs*) (n = 15–14–16–14) (**B**); ribosomal protein, large, P0 (*Rplp0*) (n = 15–14–17–14) (**C**); actin, beta (*Actb*) (n = 15–15–17–14) (**D**); and the geometric mean of all four analyzed reference genes, i.e., *Hprt, Hmbs, Rplp0, Actb* (n = 15–14–17–14) (**E**). The data are presented as the means + SDs. Each data point in the dot plot represents one individual mouse sample. Two-way ANOVA followed by Tukey's *post hoc* test (**A, D** and **E**), Welch ANOVA followed by a Games–Howell *post hoc* test (**B**) and the Kruskal–Wallis (KW) test followed by Dunn's *post hoc* test were used (**C**). Statistically significant changes ($p < 0.05$) are plotted on the graphs; *ns*, not statistically significant.

Supplemental Figure S3

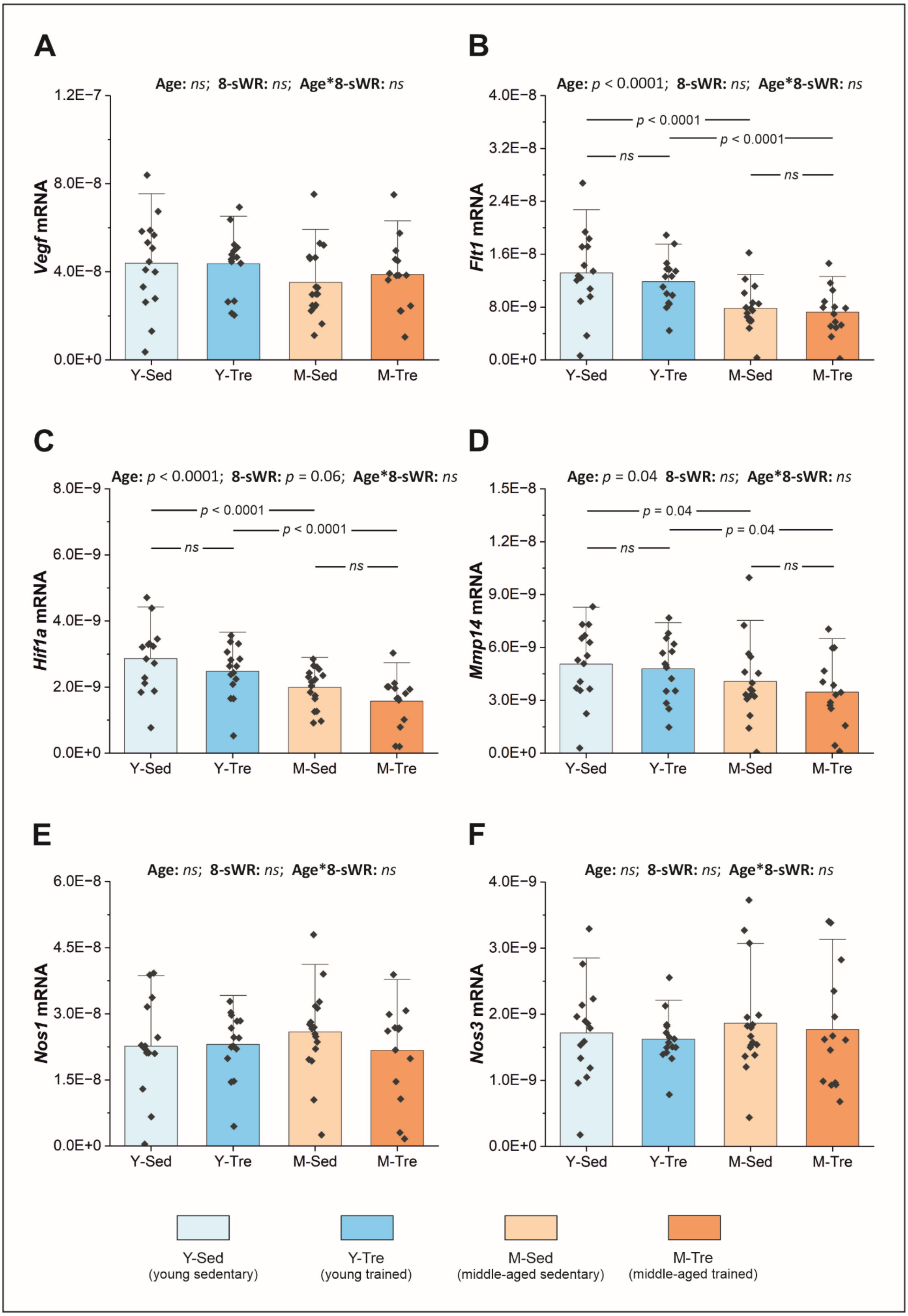

Supplemental Figure S3. Pro-angiogenic genes expression (raw data) in the fast-twitch extensor digitorum longus (EDL) muscle in the sedentary and trained groups of young (Y) and middle-aged (M) mice. The mRNA expression of vascular endothelial growth factor A (*Vegfa*) (n = 15–15–17–14) (**A**); FMS-like tyrosine

kinase 1 (*Flt1*) (n = 15–15–17–14) (**B**); hypoxia-inducible factor 1, alpha subunit (*Hif1a*) (n = 14–15–17–14) (**C**); matrix metallopeptidase 14 (*Mmp14*) (n = 15–15–16–14) (**D**); nitric oxide synthase 1 (*Nos1*) (n = 15–15–17–14) (**E**); nitric oxide synthase 3 (*Nos3*) (n = 15–15–17–14) (**F**). The data are presented as the means + SDs. Each data point in the dot plot represents one individual mouse sample. Two-way ANOVA followed by Tukey's *post hoc* test was used. Statistically significant changes ($p < 0.05$) are plotted on the graphs; *ns*, not statistically significant.

Supplemental Figure S4

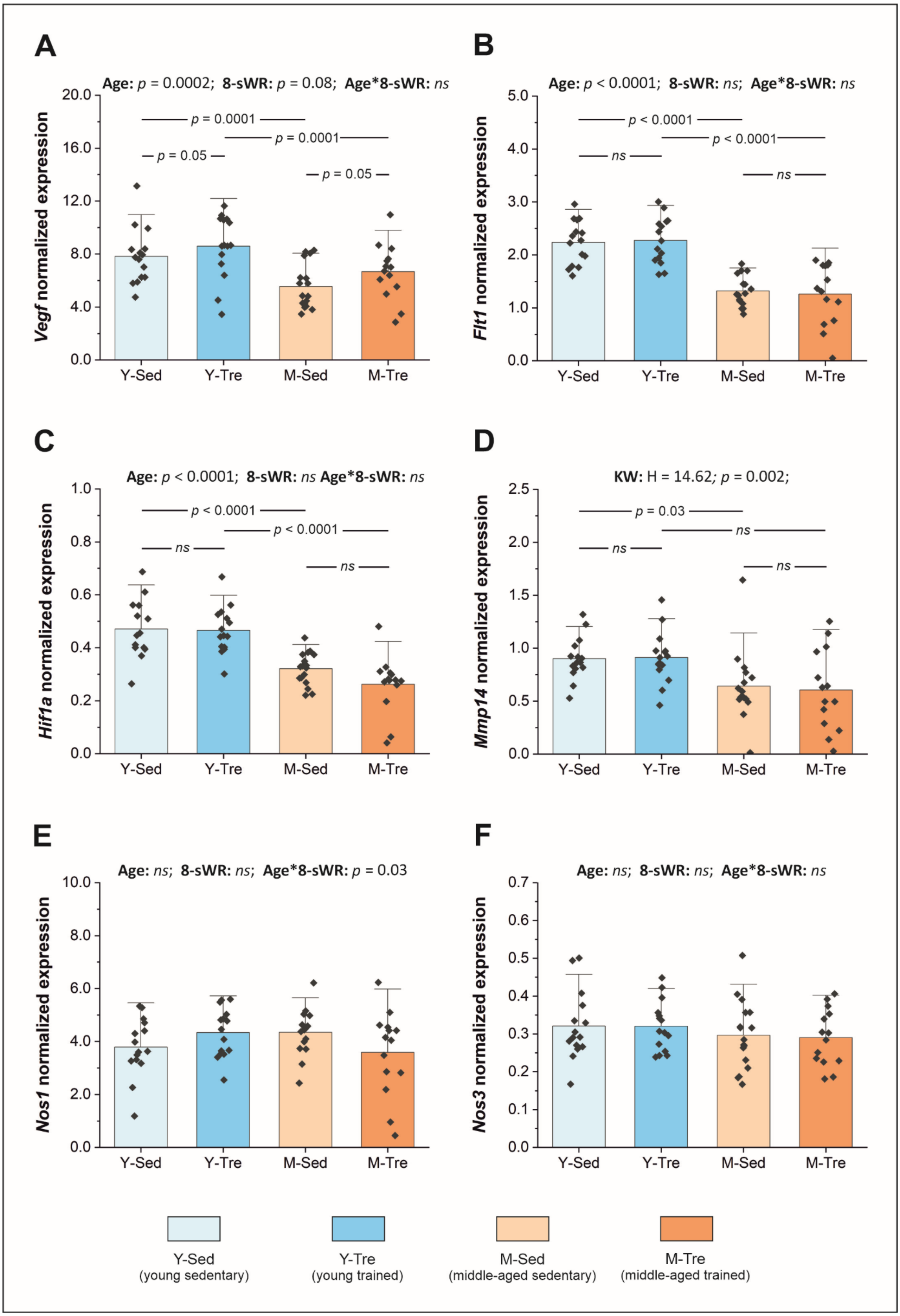

Supplemental Figure S4. Pro-angiogenic genes expression (normalized to *Hprt*) in the fast-twitch extensor digitorum longus (EDL) muscle in the sedentary and trained groups of young (Y) and middle-aged (M) mice. The expression of vascular endothelial growth factor A (*Vegfa*) (n = 15–15–17–14) (**A**); FMS-like tyrosine kinase 1 (*Flt1*) (n = 15–15–16–14) (**B**); hypoxia-inducible factor 1, alpha subunit (*Hif1a*) (n = 14–15–17–14) (**C**); matrix metallopeptidase 14 (*Mmp14*) (n = 15–15–16–14) (**D**); nitric oxide synthase 1 (*Nos1*) (n = 15–15–16–14) (**E**); nitric oxide synthase 3 (*Nos3*) (n = 15–15–17–14) (**F**). The data are presented as the means + SDs. Each data point in the dot plot represents one individual mouse sample. Two-way ANOVA followed by Tukey's *post hoc* test (**A–C**, **E** and **F**) and the Kruskal–Wallis (KW) test followed by Dunn's *post hoc* test were used (**D**). Statistically significant changes ($p < 0.05$) are plotted on the graphs; *ns*, not statistically significant.

Supplemental Figure S5

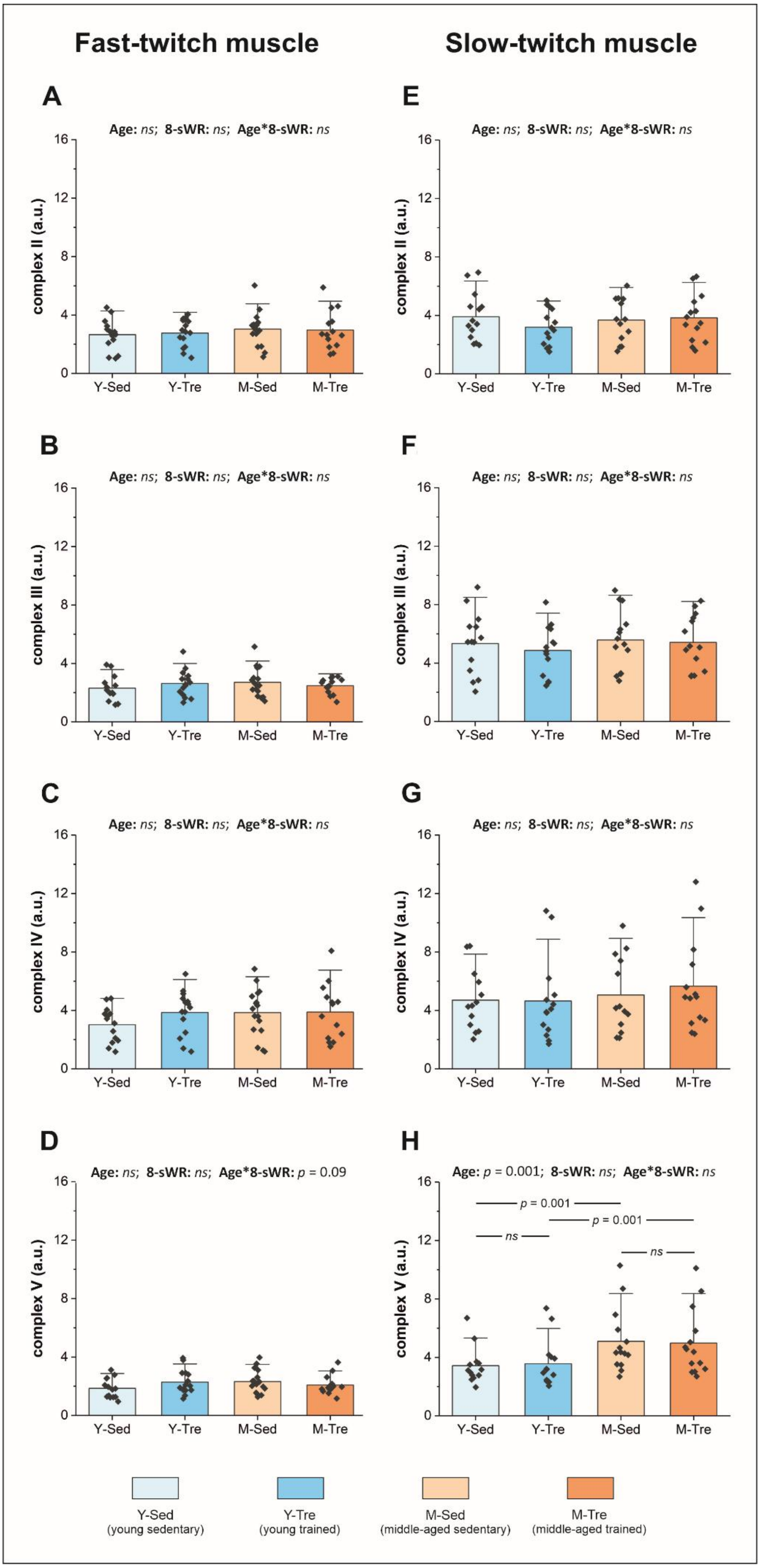

Supplemental Figure S5. Electron transport chain (ETC) proteins in the fast-twitch tibialis anterior (TA) and slow-twitch soleus (Sol) muscles in the sedentary and trained groups of young (Y) and middle-aged (M) mice. The contents of complex II (n = 14–15–17–14) (**A**), complex III (n = 14–15–17–14) (**B**), complex IV (n = 14–15–17–14) (**C**), and complex V (n = 14–15–17–14) (**D**) in the TA muscle. The contents of complex II (n = 14–14 –13 –14) (**E**), complex III (n = 14–14–14–14) (**F**), complex IV (n =13–14–13–14) (**G**), and complex V (n = 13–14–14–14) (**H**) in the Sol muscle. The data are presented as the means + SDs. Each data point in the dot plot represents one individual mouse sample. Two-way ANOVA with repeated measures followed by Tukey's *post hoc* test was used. Statistically significant changes ($p < 0.05$) are plotted on the graphs; *ns*, not statistically significant.

Supplemental Figure S6

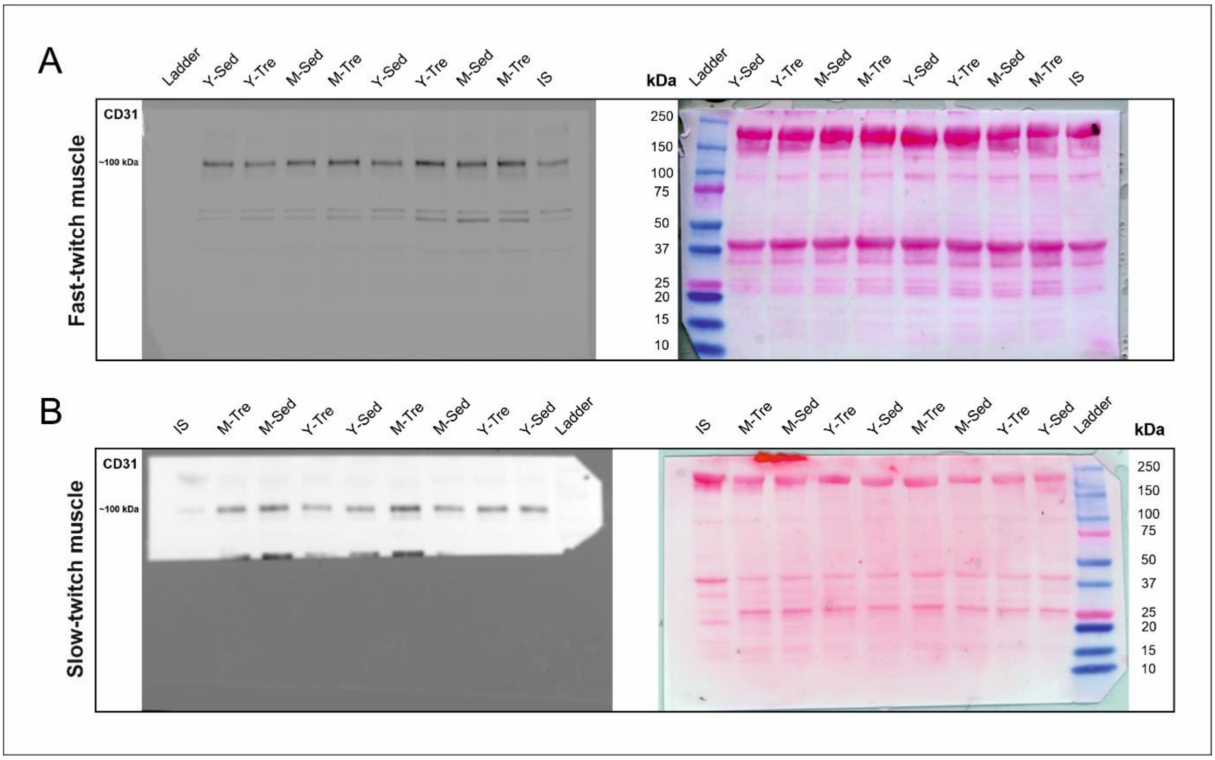

Supplemental Figure S6. Representative immunoblots demonstrating the detection of platelet endothelial cell adhesion molecule (CD31) protein in the fast-twitch tibialis anterior (TA) and slow-twitch soleus (Sol) muscles in sedentary and trained groups of young (Y) and middle-aged (M) mice. CD31 content in the TA (**A**) and Sol muscles (**B**). Ponceau S staining (right side) of the same membranes demonstrating total protein loaded. The protein ladder is a visible Precision Plus Protein Dual Color Standards (Bio-Rad, Cat# 1610374). The internal standard (IS) was a gastrocnemius sample from a wild-type mouse. ***Abbreviations:*** Y-Sed, young sedentary; Y-Tre, young trained; M-Sed, middle-aged sedentary; M-Tre, middle-aged trained.

Supplemental Figure S7

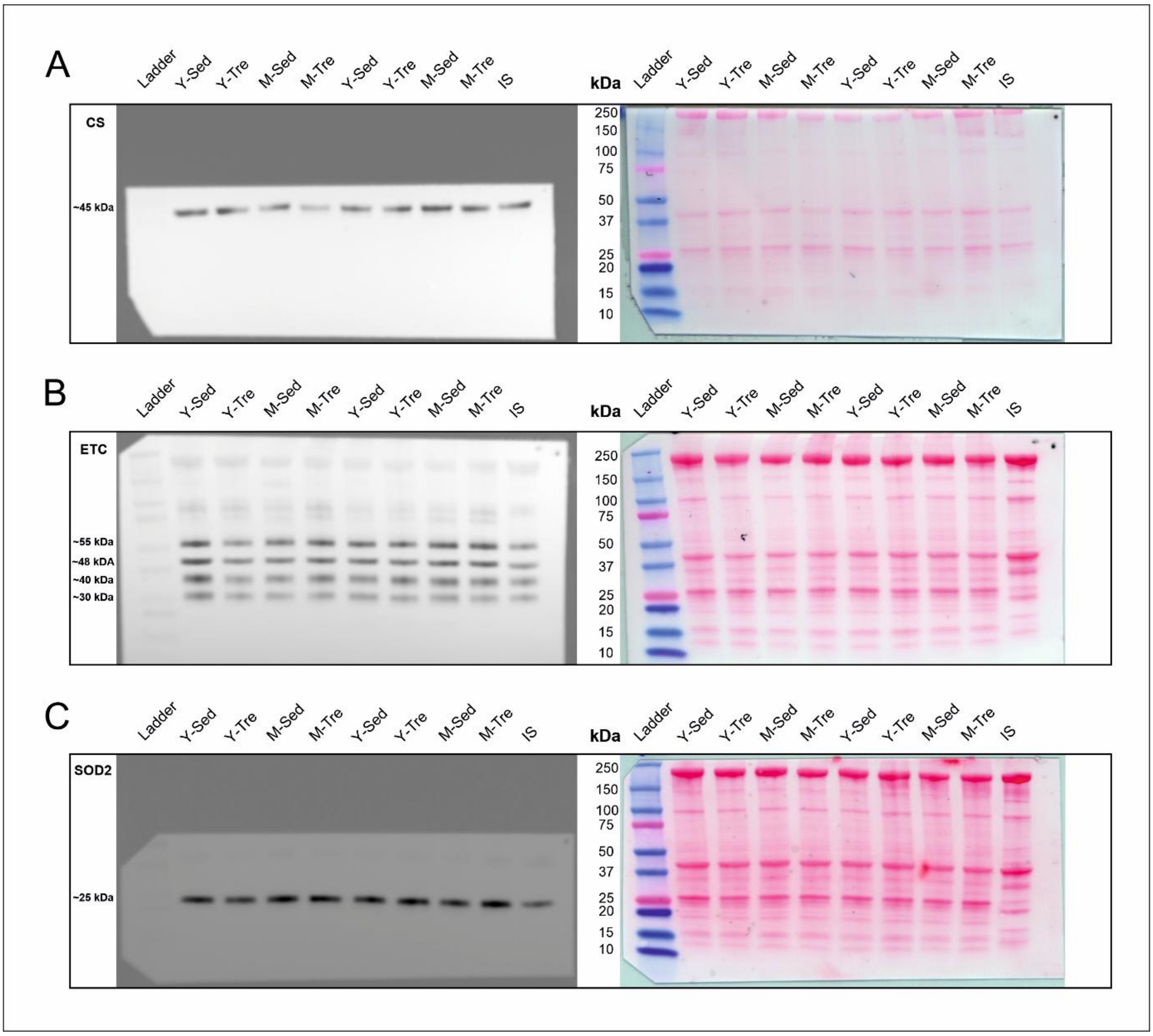

Supplemental Figure S7. Representative immunoblots showing the detection of mitochondrial protein expression in the fast-twitch tibialis anterior (TA) muscle in the sedentary and trained groups of young (Y) and middle-aged (M) mice. Citrate synthase content (CS) (~45 kDa) (**A**)**.** The electron transport chain (ETC) proteincontents including: complex II (~55 kDa), complex III (~48 kDa), complex IV (~40 kDa), and complex V (~30 kDa) (**B**). SOD2 content (~25 kDa) (**C**). Ponceau S staining (right side) of the same membranes demonstrating total protein loaded. The protein ladder is a visible Precision Plus Protein Dual Color Standards (Bio-Rad, Cat# 1610374). The internal standard (IS) was a gastrocnemius sample from a wild-type mouse. ***Abbreviations:*** Y-Sed, young sedentary; Y-Tre, young trained; M-Sed, middle-aged sedentary; M-Tre, middle-aged trained.

Supplemental Figure S8

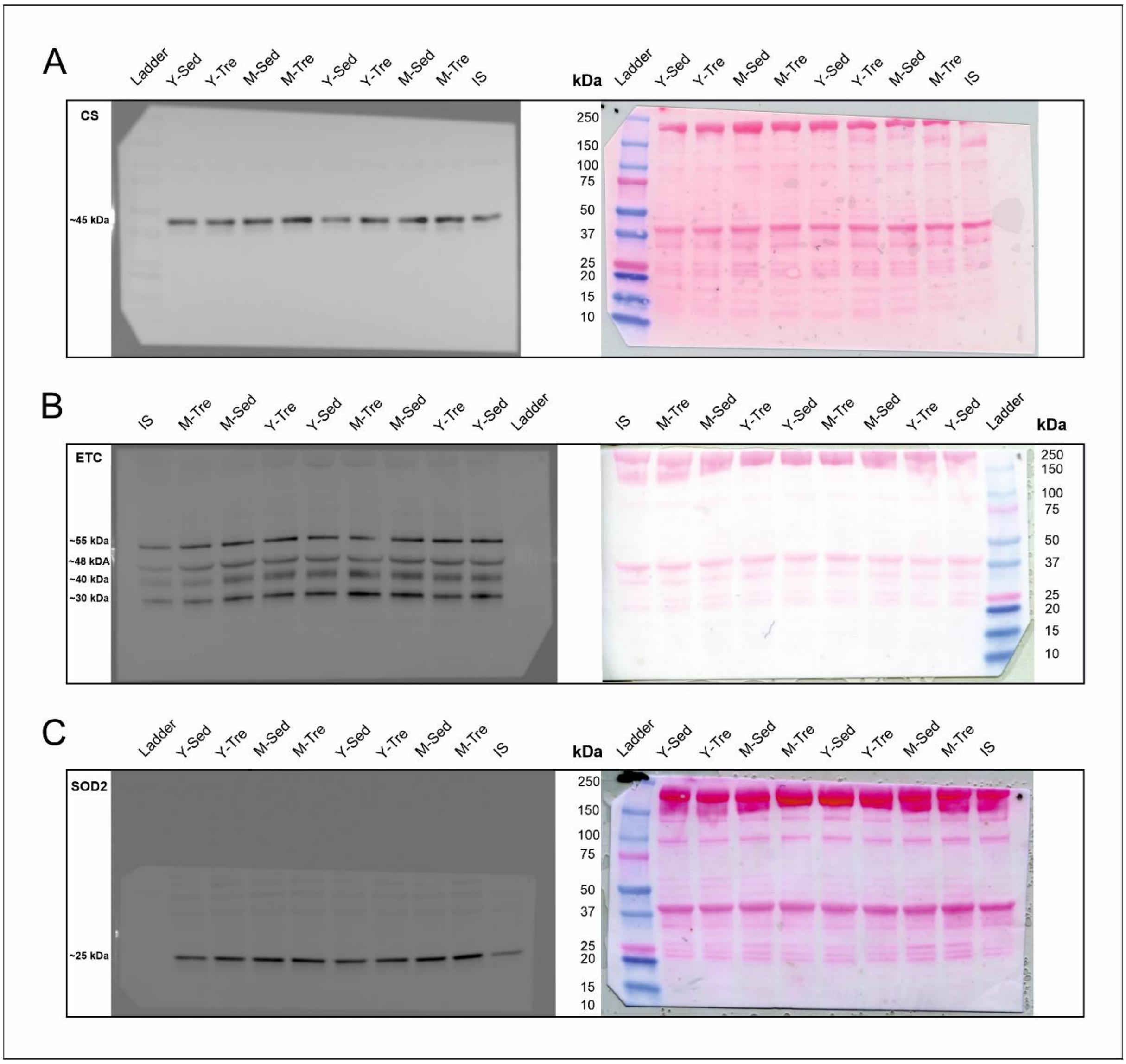

Supplemental Figure S8. Representative immunoblots demonstrating showing the detection of mitochondrial protein expression in the slow-twitch soleus (Sol) muscle in the sedentary and trained groups of young (Y) and middle-aged (M) mice. Citrate synthase content (CS) (~45 kDa) (**A**)**.** The electron transport chain (ETC) protein contents including: complex II (~55 kDa), complex III (~48 kDa), complex IV (~40 kDa), complex V (~30 kDa) (**B**). SOD2 content (~25 kDa) (**C**). Ponceau S staining (right side) of the same membranes demonstrating total protein loaded. The protein ladder is a visible Precision Plus Protein Dual Color Standards (Bio-Rad, Cat# 1610374). The internal standard (IS) was a mouse soleus (**A**) or gastrocnemius (**B** and **C**) sample from a wild-type mouse. ***Abbreviations:*** Y-Sed, young sedentary; Y-Tre, young trained; M-Sed, middle-aged sedentary; M-Tre, middle-aged trained.
